# Modulation of gene expression in immune-related organs by *in ovo* stimulation with probiotics and prophybiotics in broiler chickens

**DOI:** 10.1007/s13353-024-00891-y

**Published:** 2024-07-11

**Authors:** Ramesha N. Wishna-Kadawarage, Katarzyna Połtowicz, Rita M. Hickey, Maria Siwek

**Affiliations:** 1 Department of Animal Biotechnology and Genetics, Faculty of Animal Breeding and Biology, Bydgoszcz University of Science and Technology, Mazowiecka 28, 85-084 Bydgoszcz, Poland; 2 Department of Poultry Breeding, National Research Institute of Animal Production, Krakowska 1, 32-083 Balice, Poland; 3 Teagasc Food Research Centre, Moorepark, P61 C996 Fermoy, Co. Cork Ireland

**Keywords:** Cecal tonisls, *Leuconostoc mesenteroides*, Liver, Spleen

## Abstract

In ovo stimulation has been studied intensively as an alternative to antibiotic use in poultry production. We investigated the potential use of a probiotic in combination with a phytobiotic as a prophybiotic for in ovo stimulation and reported its beneficial effects on the gut microbiome of broiler chickens. The current study further investigates the gene expression in the immune-related organs of these chickens to understand the tissue-specific immunomodulatory effects of the treatments. The selected prophybiotic (*Leuconostoc mesenteroides* with garlic aqueous extract) and its probiotic component alone were injected into ROSS308 chicken eggs on the 12th day of incubation, and gene expression in cecal tonsils, spleen, and liver at 35 days of age was determined using qPCR method. The relative expression of each treatment was compared to the positive control, chickens injected with physiological saline in ovo. The results displayed a downregulation of pro- and anti-inflammatory cytokines in the cecal tonsils of the probiotic group and the liver of the prophybiotic group. The spleen displayed upregulated *AVBD1* in both groups and upregulated *IL1-β* in the probiotic group. The probiotic group displayed increased expression of genes related to metabolism of energy (*COX16*), protein (*mTOR*), and lipids (*CYP46A1*) whereas the prophybiotic group displayed reduced expression of genes related to cholesterol synthesis (*SREBP1*) and glucose transportation (*SLC2A2*) in the liver. In conclusion, *Leuconostoc mesenteroides* differentially modulated gene expression in chickens when administered in ovo in combination with garlic aqueous extract. Further in ovo studies with different prophybiotic combinations are required to optimize the benefits in broiler chickens.

## Introduction

The immune system of chickens consists of two main types of organs, namely primary and secondary immune organs. Primary immune organs (thymus and bursa of Fabricius) mainly function in the production and maturation of the immune cells whereas secondary immune organs including the spleen and cecal tonsils are involved in the activation of immune cells (Wlaźlak et al. 2023). Although the development of the immune system in chickens starts during the embryonic development stage, the functional capacity of these organs in neonatal chicks is limited, as the most immune cells are immature at the time. It takes approximately 2 weeks to gain functional maturity of their immune system (Slawinska et al. 2014) whereas the maternal antibodies in neonates fade away with the time post-hatching (Alizadeh et al. 2017). Therefore, during the early post-hatch period (until the age of 2–3 weeks), the chicks remain vulnerable to many pathogens. Therefore, many research groups focus on the potential of early activation of the immune system via different in ovo interventions.

Consequently, in ovo administration of probiotics has been identified as one of the promising methods in modulating the immune system of chickens. Probiotics have been shown to induce the production of cytokines by activating the pattern recognition receptors on immune cells thereby regulating the immune responses (Alizadeh et al. 2017). Probiotics are also known to stimulate the production of natural antibodies in chickens (Haghighi et al. 2006). Moreover, in ovo administration of probiotics increased macrophage counts in the spleen and the antibody-mediated immune response in chickens (Alizadeh et al. 2021). Similarly, phytobiotics (plant-derived bioactives) display potential in modulating the immune system of chickens upon in ovo administration*.* Previously, El-Kholy et al. (2021) reported that in ovo administration of plant extracts (cinnamon, thyme, and clove) resulted in an increased serum concentration of immunoglobulins G and M in adult chickens. Although the immunostimulation effects of dietary supplementation of phytobiotics have been demonstrated widely (Akosile et al. 2023), in ovo administration effects have not been sufficiently investigated.

To the best of our knowledge, our research group reported for the first time the potential use of a probiotic and phytobiotic in combination (a prophybiotic) for in ovo stimulation (Wishna-Kadawarage et al. 2024b). For this application, *Leuconostoc mesenteroides* B/00288 probiotic strain was selected as it displayed significant antimicrobial activity against multiple *Salmonella enterica* strains and *Campylobacter jejuni* (Wishna-Kadawarage et al. 2024a)*.* For the phytobiotic component, garlic aqueous extract was used, as it does not inhibit the growth of *L. mesenteroides* B/00288 at a concentration of 0.5% as shown previously (Wishna-Kadawarage et al. 2023). In ovo stimulation with the selected prophybiotic or its probiotic component alone resulted in no adverse effects on hatchability, chick quality, and production parameters in ROSS 308 broiler chickens (Wishna-Kadawarage et al. 2024b). Moreover, the treatments resulted in beneficial effects on the gut microbiome and prophylactic effects on histomorphometry and gene expression in the ceca where most of the gut microbiome is located in chickens.

However, the activation of the immune system can be a double-edged sword as it leads to a divergence of energy from production during the process of eliminating the pathogens (Korver 2006). It is also worth highlighting that there is a significant interplay between inflammation and metabolic reactions (Roche 2021). An inappropriate supplementation or intervention could lead to inflammation in the gut thereby affecting metabolism. However, in these in ovo stimulated chickens, no impairment of the production parameters was observed although an activation of the immune system was displayed in the ceca indicating no significant immune-metabolism tradeoff resulting from these in ovo treatments. Therefore, the current study was performed to further investigate the tissue-specific immune modulation and metabolic gene regulation of these in ovo stimulated chickens. Accordingly, the expression of immune-related genes was determined in the secondary immune organs, cecal tonsils, and the spleen as well as the liver which is an important organ for both the immune system and metabolism. Additionally, the expression of the genes related to metabolism was analyzed in the liver.

## Materials and methods

### Experimental design

The current study is a continuation of the animal experiment reported in Wishna-Kadawarage et al. (2024b). Three experimental groups, namely, positive control (PC), probiotic (PB), and prophybiotic (PPB), were included in the current study. The in ovo injections provided to the birds of each group are described in Table 1.

**Table 1:** Description of in ovo injections given to experimental groups

Experimental group	In ovo injection	Dose	Volume
Positive control (PC)	0.9% NaCl physiological saline solution	None	0.2 mL
Probiotic (PB)	*Leuconostoc mesenteroides* B/00288	10^6^ CFU/egg	0.2 mL
Prophybiotic (PPB)	*Leuconostoc mesenteroides* B/00288 + Garlic aqueous extract (in 2:1 ratio of total volume)	LM: 10^6^ CFU/egg Garlic: 0.5% (w/v)	0.2 mL

### In ovo stimulation protocol

In ovo stimulation was performed as previously described in Wishna-Kadawarage et al. (﻿2024b). Briefly, incubation of ROSS 308 hatching eggs (100 eggs/group) was carried out under the standard conditions (temperature of 37.5 °C and relative humidity of 55%) using an automated incubator (Midi series, Fest Incubators, Poland). On the 12th day of incubation, eggs were randomly allocated into different in ovo treatments and received the respective injections as described in Table 1. After disinfecting the eggshell with 70% ethanol, all injections were performed manually at the site of the air cell without damaging the membranes. Egg injections were performed as quickly as possible, and eggs were returned to the incubator to complete the incubation process.

### Preparation of PB injection

The probiotic strain *L. mesenteroides* was cultured in MRS broth (BD Difco 288,130, Fisher Scientific, Ireland) for 15 h at 37 °C to prepare the probiotic inoculum for in ovo administration. The culture was then centrifuged at 4200 g for 20 min at 4 °C to obtain the bacterial pellet. Sterile 0.9% NaCl physiological saline solution (Natrium Chloratum 0.9% Fresenius KabiPac, Fresenius Kabi, Poland) was used to re-suspend the bacterial pellet adjusting the optical density at 600 nm (OD600) to 5 × 10^6^ CFU/mL. A 0.2 mL (corresponding to 10^6^ CFU/egg) aliquot was injected into each egg in the PB group from this suspension.

### Preparation of PPB injection

For the PPB injection, a separate probiotic suspension in sterile physiological saline solution was prepared adjusting OD600 to 7.5 × 10^6^ CFU/mL. In addition to that, an aqueous extract (1.5% w/v) of garlic (cultivar: Thermodrome, organically grown in the 2021 season in Aarhus University, Department of Food Science at Research Centre at Årslev, Funen, Denmark) was prepared as described in Wishna-Kadawarage et al. (2023). Briefly, the garlic powder was incubated with sterile distilled water to activate the reaction of allin enzyme, producing allicin which is a well-known antimicrobial and immunomodulatory compound in garlic. The probiotic suspension and garlic aqueous extract were then mixed in a 2:1 ratio in volume, resulting in a final concentration of 10^6^ CFU of probiotic (the same probiotic dose as the PB group) and 0.5% (w/v) garlic aqueous extract when 0.2 mL of the mixture was administered to each egg.

### Animal experiment and sample collection

The animal experiment was carried out as described by Wishna-Kadawarage et al. (2024b), in accordance with the guidelines of the Ethics Committee for Experiments with Animals and regulations of the Polish Act on the Protection of Animals Used for Scientific or Educational Purposes of 15 January 2015. Briefly, upon hatching, chickens were transported and housed in groups on deep litter floor pens (one pen/in ovo treatment group) which were electronically controlled to provide uniform conditions. The chickens were raised until 35 days, and eight birds per group were sacrificed (by decapitation after 10 h of fasting) and immune-related tissues, namely, cecal tonsils, spleen, and liver, were collected. All samples were cut into small pieces and were transported in tubes containing fix RNA stabilization buffer (E0280, EURx, Poland) at room temperature. The samples (excluding fix RNA buffer) were frozen at − 80 °C until processed.

### Gene expression analysis in immune-related tissues

#### RNA extraction

Approximately, 300 mg of each tissue sample was homogenized with 1 mL of RNA Extracol solution (E3700, EURx, Poland) using a TissueRuptor II homogenizer (990,890, Qiagen, Poland). Next, 0.2 mL of chloroform (112,344,305, Chempur, Poland) was added to the tissue homogenate, and this was centrifuged (at 12,000 g for 15 min at 4 °C) to isolate RNA in the supernatant which was further purified using the Universal RNA purification kit (E3598, EURx, Poland) according to the manufacturer’s protocol. The quality and quantity of the RNA were determined using the NanoDrop 2000 spectrophotometer (Thermo Scientific, Poland) while RNA integrity was confirmed via gel electrophoresis (2% agarose gel). The RNA samples were then frozen at − 80 °C until subsequent use.

#### Quantitative reverse transcription PCR (RT-qPCR)

Gene expression analysis was performed using RT-qPCR using two steps. First, reverse transcription of RNA was performed using a smART First Strand cDNA Synthesis Kit (0804, EURx, Poland) following the manufacturer’s protocol. Second, qPCR was performed with a reaction mixture of 12.5 µL containing 20 ng of cDNA, 1 µM each of forward and reverse primers (Sigma-Aldrich, Germany), and 6.25 µL of SG qPCR Master Mix (2 ×) (0401, EURx, Poland). Two technical replicates of each qPCR reaction were performed in 96 well plates (4TI-0955, AZENTA, Poland). Thermo-cycling protocol involved a pre-incubation step (at 95 °C for 15 min) with 40 cycles of subsequent denaturation (95 °C for 15 s), annealing (58˚C for 30 s), and elongation (72 °C for 30 s) steps using a LightCycler 480 II (Roche-Diagnostics, Switzerland). The average Ct values of the technical replicates were used to calculate the relative gene expression of the selected genes using the ΔΔCt method (Livak and Schmittgen 2001). The details of the genes selected for analysis along with their primer sequences are outlined in Table 2

**Table 2:** Primers used for the qPCR to determine the relative gene expression in cecal mucosa

Gene name	Gene symbol	Primer sequence^1^ (5′→3′)	Reference
House-keeping genes			
Actin, beta	*ACTB*	F: CACAGATCATGTTTGAGACCTT	Sevane et al. (2014)
		R: CATCACAATACCAGTGGTACG	
Glucose-6-Phosphate Dehyfrogenase	*G6PDH*	F: CGGGAACCAAATGCACTTCGT	Sevane et al. (2014)
		R: GGCTGCCGTAGAGGTATGGGA	
Immune-related genes			
Avian beta-defensin 1	*AVBD1*	F: AAACCATTGTCAGCCCTGTG	Slawinska et al. (2019)
		R: TTCCTAGAGCCTGGGAGGAT	
Cathelicidin 2	*CATHL2*	F: AGGAGAATGGGGTCATCAGG	Slawinska et al. (2019)
		R: GGATCTTTCTCAGGAAGCGG	
Free fatty acid receptor 2	*FFAR2*	F: GCTCGACCCCTTCATCTTCT	Slawinska et al. (2019)
		R: ACACATTGTGCCCCGAATTG	
Interleukin 1 beta	*IL1-β*	F: GGAGGTTTTTGAGCCCGTC	Dunisławska et al. (2017)
		R: TCGAAGATGTCGAAGGACTG	
Interleukin 2	*IL2*	F: GCTTATGGAGCATCTCTATCATCA	Pietrzak et al. (2020)
		R: GGTGCACTCCTGGGTCTC	
Interleukin 6	*IL6*	F: AGGACGAGATGTGCAAGAAGTTC	Chiang et al. (2009)
		R: TTGGGCAGGTTGAGGTTGTT	
Interleukin 8	*IL8*	F: AAGGATGGAAGAGAGGTGTGCTT	Slawinska et al. (2014)
		R: GCTGAGCCTTGGCCATAAGT	
Interleukin 10	*IL10*	F: CATGCTGCTGGGCCTGAA	Rothwell et al. (2004)
		R: CGTCTCCTTGATCTGCTTGATG	
Metabolic genes			
Cytochrome C Oxidase Assembly Factor COX16	*COX16*	F: CCTGCTTTGAAGGAAAAATTGAAG	Criado-Mesas et al. (2021)
		R: CCAAGTCAGATTGTTCCAATTTCTC	
Sterol regulatory element-binding protein-1	*SREBP1*	F: GAGGAAGGCCATCGAGTACA	Dridi et al. (2005)
		R: GGAAGACAAAGGCACAGAGG	
Cytochrome P450 Family 46 Subfamily A Member 1	*CYP46A1*	F: CATGCCGCGTATGACCACAT	Idriss et al. (2018)
		R: GCCATTCCCCAGAAACCTCAGA	
Mechanistic target of rapamycin	*mTOR*	F: TGCTGACAAACGCTATGGAGGT	Criado-Mesas et al. (2021)
		R: AGCCATGACACTGTCCTTATGCT	
Glucose-6-phosphatase catalytic subunit	*G6PC*	F: GCGTCTGGTATGTAATGG	Guo et al. (2015)
		R: AGAATAACTTGATGAGGGA	
Solute Carrier Family 2 Member 2	*SLC2A2*	F: GAGGAGGCCAAAAAGAGTTTG	Criado-Mesas et al. (2021)
		R: ACTCTCTTTTCACTCGCAGCTTCT	

^1^*F* forward primer, *R* reverse primer

### Statistical analysis of data

After removing the outliers (values which are greater than Quartile 3 + 1.5 × interquartile range and below Quartile 1 + 1.5 × interquartile range) of the ΔCt values of the samples, the mean of each treatment group was compared to the mean of the PC group using two sample *T*-test in R (version 4.3.1) to identify statistical significance (*P-*value < 0.05) or tendency (*P*-value < 0.1) of the changes in relative expression of the selected genes as a result of each treatment. In cases where the data were not normally distributed, the Wilcoxon rank-sum test was used to identify significant gene expression changes in the treatment group compared to the PC group.

## Results and discussion

In the current study, gene expression profiling was performed to analyze the tissue-specific immunomodulation and metabolic regulation of adult broiler chickens who were subjected to in ovo stimulation with a selected prophybiotic combination (*Lecuconostoc mesenteroides* + garlic aqueous extract) and the probiotic component alone. A panel of immune-related genes were selected for this study and included genes encoding for pro- and anti-inflammatory cytokines: *IL1-β*, *IL2*, *IL6*, and *IL10*; pro-inflammatory chemokine: *IL8*; free fatty acid receptor 2 (*FFAR2*); and host defense peptides: *AVBD1* and *CATHL2*. The relative expression of the genes was analyzed in secondary immune organs, cecal tonsils and spleen and the liver, which plays a dual role in the immune system and metabolism. The panel of metabolic genes included *COX16*, *SREBP1*, *CYP46A1*, *mTOR*, *G6PC*, and *SLC2A2*, and their expression was analyzed in the liver. The results revealed an organ-specific pattern of immunomodulation and metabolic regulation in the liver resulting from the prophybiotic and probiotic in ovo stimulations.

### Cecal tonsils

**Fig. 1:**
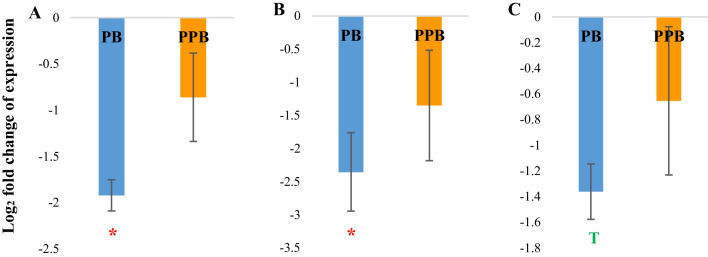
The expression of immune-related genes in the cecal tonsils of chickens (*n* = 8) treated with PB (probiotic) (*Leuconostoc mesenteroides*) and PPB (prophybiotic) (*Leuconostoc mesenteroides* + garlic aqueous extract) in ovo, relative to the expression of chickens of the control group*.* **A** *IL1B*, **B** *IL8*, **C** *IL10*. Error bars: ± SE. Red color asterisk (*) indicates significant changes (*P-*value < 0.05). The letter T in green indicates there is a tendency (*P-*value < 0.1)

The cecal tonsils are the largest gut-associated lymphoid tissues in chickens (Zhang et al. 2023). In the cecal tonsils, the probiotic treatment resulted in a significant downregulation of *IL1-β*, *IL8*, and *IL10* genes whereas the prophybiotic group did not show a significant difference in the expression of any of the immune-related genes studied, when compared to the positive control group (Fig. 1).

The chicken *IL1-β* gene encodes a protein (cytokine) which is secreted from leucocytes and is important in a pro-inflammatory immune response by supporting T- and B-cell proliferation, secretion of other immune molecules, and increasing the expression of cytokine receptors (Lee et al. 2014). *IL1-β* gene is usually over-expressed during inflammation or infections by organisms such as *Eimeria* (Wigley and Kaiser 2003) and *Salmonella* (Zhang et al. 2023). *IL8* moreover is a pro-inflammatory chemokine which attracts neutrophils to the site of inflammation during infection (Kogut 2002; Elnagar et al. 2021). Therefore, the downregulation of pro-inflammatory genes *IL1-β* and IL8 in the cecal tonsils by the probiotic treatment may indicate enhanced immunotolerance to the local microbiome as described by Rubio (2019). As Slawinska et al. (2016) reported, an acquired immunotolerance to a healthy microbiome will support the colonization of beneficial bacteria eventually mitigating the colonization of pathogens in the gut. Moreover, Pender et al. (2017) claimed that the downregulation of immune-related genes in cecal tonsils of adult chickens which were treated with probiotics in ovo could be a consequence of reduced pathogenic load and virulence or increased clearance of pathogens due to the acquired healthy microbiome. Complementary to our results, Slawinska et al. (2016) and Alizadeh et al. (2020) observed a downregulation of pro-inflammatory cytokine expression in the cecal tonsils following in ovo administration of synbiotics and probiotics, respectively, in chicken.

In contrast, *IL10* is an anti-inflammatory cytokine which inhibits the activity of pro-inflammatory cytokines such as *IL1-β* and helps in preventing cell damage caused by inflammation and maintaining the immune homeostasis in the host (Lee et al. 2018). As an anti-inflammatory response usually follows a pro-inflammatory response and pro-inflammatory molecules were downregulated in the cecal tonsils, it is possible that the downregulation of *IL10* (anti-inflammatory) is an adaptation of the immune system in the probiotic group.

However, it is interesting that the prophybiotic treatment containing the same probiotic species did not cause any change to the expression of the immune-related genes in the cecal tonsils when compared to the PC group. Garlic aqueous extract may contain fructans which can act as a prebiotic (Zhang et al. 2013). Thus, it is very likely that the garlic component of the treatment modulated the gut microbiome different from the probiotic alone treatment possibly requiring no such immunotolerance given a different microbiome composition. In agreement, we previously reported some changes observed in the cecal microbiome of these in ovo stimulated birds (Wishna-Kadawarage et al. 2024b). In the cecal content of these birds, the prophybiotic treatment resulted in a reduction of *Escherichia coli* and *Faecalibacteria* species and an increase in *Akkermansia* sp. was observed in both prophybiotic- and probiotic-treated birds when compared to the positive control group.

Another possibility is that the garlic extract recruited more immune cells in the cecal tonsils which eventually produce pro-inflammatory cytokines (Arreola et al. 2015) in parallel to the immune tolerance acquired due to the probiotic used in combination. Further studies on the exact chemical composition of the garlic aqueous extract might provide us important clues about this possibility. Either way, it seems that the prophybiotic treatment resulted neither in an extra activation nor a deactivation of the immune genes when compared to the positive control. Overall, the probiotic group demonstrated a metabolic benefit as it reduced the cost of maintaining immunity whereas the prophybiotic treatment demonstrated more of an immunological benefit as it did not decrease the level of the immunotolerance during the process of early stimulation of the gut microbiome.

### Spleen

**Fig. 2:**
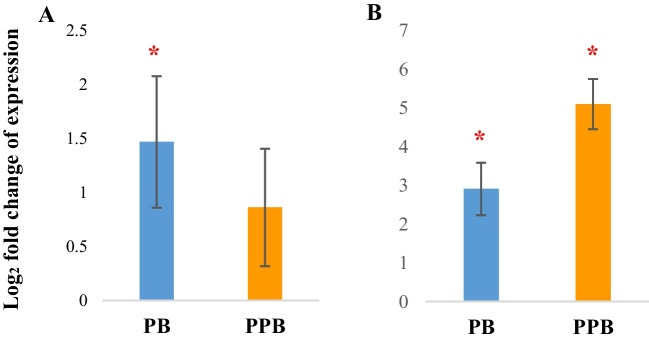
The expression of immune-related genes in the spleen of chickens (*n* = 8) treated with PB (probiotic) (*Leuconostoc mesenteroides*) and PPB (prophybiotic) (*Leuconostoc mesenteroides* + garlic aqueous extract) in ovo, relative to the expression of chickens of the control group*.* **A** *IL1B*, **B** *AVBD1*. Error bars: ± SE. Red color asterisk (*) indicates significant changes (*P-*value < 0.05)

Representing the largest peripheral lymphoid organ in chicken, the spleen plays a major role in the immune system of chickens by filtering blood via the recruitment of immune cells, which acts as a barrier to blood-borne pathogens (Zhang et al. 2015). In our study, we observed an upregulation of *AVBD1* gene expression in the spleen of both probiotic and prophybiotic treated chickens whereas the prophybiotic treatment displayed the highest fold change when compared to the positive control. In addition, the probiotic but not prophybiotic treatment resulted in an over-expression of *IL1-β* gene in the spleen (Fig. 2).

Avian defensins are small peptides which are secreted by host immune/epithelial cells and act as broad-spectrum antimicrobials via various methods such as membrane disruption and inhibition of cell wall synthesis of the pathogens (Xu and Lu 2020). Among the 14 avian defensins identified, *AVBD1* has been shown to be greatly expressed in the spleen of broiler chickens (Lyu et al. 2020) possibly as a means to destroy the pathogens filtered in the spleen. Therefore, our results suggest that in ovo treatment of both the probiotic and the prophybiotic used in the current study induces the immune system in the spleen to favor anti-pathogenic activity whereas the prophybiotic treatment provides an immunological as well as metabolic (as there was no sign of inflammation) advantage in modulating the gene expression in spleen.

In agreement with our results, it has been reported that probiotic *Lactobacillus rhamnosus* induced the expression of avian beta-defensin 9 in chicken splenocytes in vitro without increasing the expression of pro-inflammatory cytokines (Huang et al. 2020). Moreover, Brisbin et al. (2010) observed an over-expression of *IL1-β* in chicken spleen cells when co-cultured with different *Lactobacillus* strains. Several other studies observed an over-expression of other pro-inflammatory cytokines in the spleen of in ovo probiotic-treated broiler chickens (Slawinska et al. 2016; Alizadeh et al. 2020; Pietrzak et al. 2020).

### Liver

The liver is considered not only a metabolically important organ, but also an important organ in immunology as it contains a lot of immune cells and plays a role in the synthesis of complement components and other pathogen recognition receptors (Liu et al. 2021). This is mainly because the liver is exposed to a multitude of foreign antigens delivered with the hepatic portal blood coming from the gut (Robinson et al. 2016). Therefore, it is important to investigate the gene expression of the liver from both a metabolic and an immunological point of view. Interestingly, our study indicated a differential modulation of both immune and metabolism-related gene expression in the liver in response to in ovo stimulation with the probiotic and the prophybiotics.

**Fig. 3:**
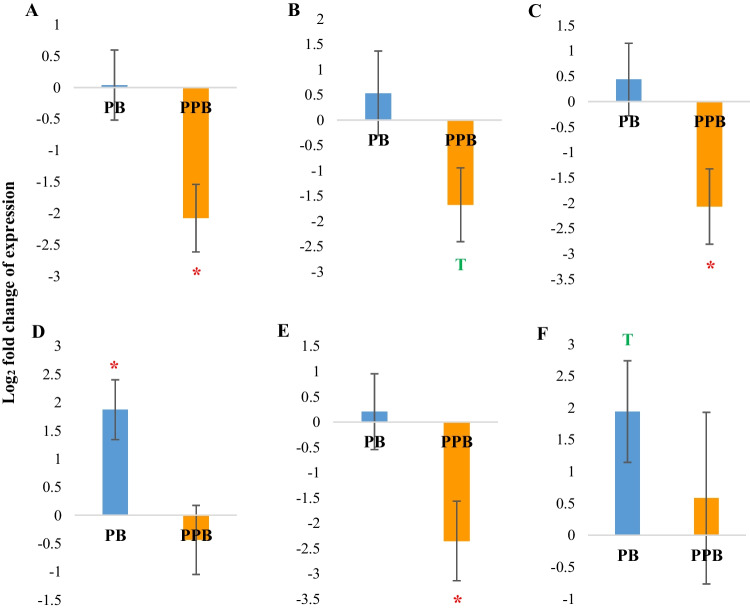
The expression of immune-related genes in the liver of chickens (*n* = 8) treated with PB (probiotic) (*Leuconostoc mesenteroides*) and PPB (prophybiotic) (*Leuconostoc mesenteroides* + garlic aqueous extract) in ovo, relative to the expression of chickens of the control group*.* **A** *IL1B*, **B** *IL2*,** C** *IL6*, **D** *IL8*,** E** *IL10*, **F** *FFAR2*. Error bars: ± SE. Red color asterisk (*) indicates significant changes (*P-*value < 0.05). The letter T in green indicates there is a tendency (*P-*value < 0.1)

The prophybiotic treatment resulted in a downregulation of both the pro-inflammatory (*IL1-β*, *IL2*, and *IL6*) and anti-inflammatory (*IL10*) cytokines studied in the liver (Fig. 3 A, B, C, and E, respectively). This indicates that the liver became more tolerant to the foreign antigens coming from the gut via the hepatic portal blood. Similarly, in a previous study, it has been shown that in ovo administration of a synbiotic resulted in a downregulation in the expression of immune-related genes in the liver of ROSS 308 broiler chickens (Dunisławska et al. 2023).

The probiotic treatment, however, increased the expression of *IL8* and *FFAR2* genes in the liver (Fig. 3D and 3F). *FFAR2* gene encodes an important receptor which performs both metabolic and immunological functions. The *FFAR2* receptor plays a major role in energy sensing (via signaling molecules such as short-chain fatty acids (SCFAs)) and in regulating carbohydrate metabolism by inducing the secretion of insulin and incretin hormones (Hara et al. 2013). It is also known to regulate the inflammatory response upon activation by microbial metabolites such as SCFAs (Akhtar et al. 2022). As the probiotic treatment caused the over-expression of this *FFAR2* gene but not the prophybiotic treatment, it is possible that the microbial metabolites that reached the liver through the hepatic portal vein were different in the probiotic group when compared to the prophybiotic group, further supporting our previous hypothesis that when administered in combination, the garlic component modifies the gut microbiome differently compared with administering the probiotic alone. *FFAR2* is also found largely in immune cells such as monocytes and B-lymphocytes (Besten et al. 2013). Thus, it is also possible the probiotic treatment recruited more immune cells in the liver. Overall, it can be suggested that the in ovo treatment of the probiotic used in the current study provides a more antimicrobial role whereas the prophybiotic induces a more antigenic tolerance in the liver of the chickens.

**Fig. 4:**
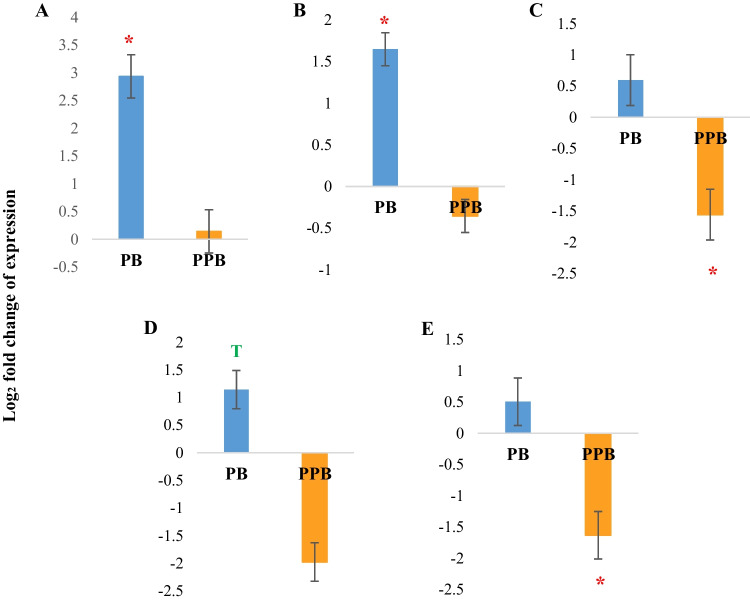
The expression of metabolic genes in the liver of chickens (*n* = 8) treated with PB (probiotic) (*Leuconostoc mesenteroides*) and PPB (prophybiotic) (*Leuconostoc mesenteroides* + garlic aqueous extract) in ovo, relative to the expression of chickens of the control group*.* **A** *COX16*, **B** *mTOR*,** C** *SREBP1*, **D** *CYP46A1*, **E** *SLC2A2.* Error bars: ± SE. Red color asterisk (*) indicates significant changes (*P-*value < 0.05). The letter T in green indicates there is a tendency (*P-*value < 0.1)

Interestingly, the analysis of metabolic gene expression in the liver also showed differential effects in response to the two treatments, probiotic and prophybiotic. The probiotic treatment resulted in an upregulation of *COX16*, *mTOR*, and *CYP6A1* genes whereas the prophybiotic treatment resulted in a downregulation of *SREBP1* and *SLC2A2* genes in the liver (Fig. 4). Both treatments did not result in any significant change in the relative expression of the *G6PC* gene.

The *COX16* gene encodes an important protein which is crucial for the last step of the oxidative phosphorylation process in mitochondria (Su and Tzagoloff 2017) and thus important in energy metabolism. The *mTOR* gene encodes one of the protein complexes in the mTOR pathway which regulates protein synthesis and cell proliferation (Mori et al. 2014) whereas *CYP46A1* plays an important role in bile synthesis via facilitating sterol efflux in the liver (Idriss et al. 2017). Over-expression of these genes in the liver may indicate a greater protein and lipid metabolism in the probiotic-treated chickens. Similar results were previously observed by Dunisławska et al. (2021) where proteomic changes indicated an accelerated metabolism in the liver upon in ovo treatment with a *Lactobacillus* synbiotic.

Prophybiotic treatment, however, displayed a possible downregulation of glucose transportation (Bae et al. 2010) (via downregulation of *SLC2A2*) as well as cholesterol biosynthesis (Horton et al. 2002) (via downregulation of *SREBP1*) in the liver. As glucose is a crucial supplier of the raw material for cholesterol biosynthesis (Xiao et al. 2022), it can be suggested that the prophybiotic treatment modulated the gene expression in the liver to reduce cholesterol production. It is a well-known paradigm that garlic exhibits hypoglycemic properties in liver cells (Chang and Johnson 1980; Liu and Yeh 2002; Xie et al. 2023) and that in ovo treatments with bioactives modulate liver gene expression via epigenetic pathways such as gene methylation (Dunisławska et al. 2023) and microRNA mediated regulation (Sikorska et al. 2021). Moreover, it is reported that bioactive components in garlic, particularly diallyl trisulfide, play a key role in DNA methylation and histone modifications leading to epigenetic effects (Zhang et al. 2024). Therefore, it can be suggested that the garlic component in the prophybiotic treatment imparted a long-term effect on the expression of genes related to cholesterol synthesis in the liver possibly via epigenetic regulation.

Taken together, the results of gene expression analysis (both immune-related and metabolic genes) in the liver indicate that the probiotic used in the current study induced metabolic functions as well as inflammatory response whereas the prophybiotic resulted in a downregulation of both inflammatory cytokines as well as cholesterol synthesis in the liver. As cholesterol accumulation is highly positively correlated to the inflammatory response in the liver (Mueller et al. 2021), it is possible that the hypoglycemic effects imparted from the garlic components in the prophybiotic treatment resulted in the under-expression of the inflammatory cytokines in the liver. Interestingly, although the current study indicated that genes related to lipid and protein metabolism were differentially modulated, previously reported results indicated that there were no significant differences in the body weight and the fat composition of the carcasses among different groups (Wishna-Kadawarage et al. 2024b). Therefore, it can be suggested that the metabolic regulation resulting from these treatments was not influencing the production of the animal, but instead maintaining the homeostasis to adapt with immunomodulation changes. According to our results, in probiotic-treated chickens, it is possible that the energy burden conferred by upregulating immune parameters in the liver and the spleen was compensated by the metabolic benefit gained from upregulating metabolism in the liver and downregulating immune parameters in cecal tonsils. The immune parameters of the prophybiotic group, however, displayed an upregulation in the spleen and a downregulation in the liver, indicating a possible compensation of energy burden in immunomodulation without affecting the body weight.

However, a limitation of the current study is that the gene expression has been evaluated at the mRNA level. It is important to note that post-transcriptional modifications may cause changes at the protein level leading to physiological effects different from the predictions made based on the mRNA results. Moreover, gene expression alone cannot be used to conclude the exact biological effect in these chickens. Further studies on the gut microbiome profiling, proteome, and metabolome are necessary to recommend whether the probiotic or the prophybiotic application can be more beneficial for the broiler chickens. In addition, the probiotic dose delivered in the current study was 10^6^ CFU/egg while some other studies displayed similar effects on immune-related gene expression by stimulating chicken eggs with different probiotics at a dose as low as 10^3^ CFU/egg (Siwek et al. 2018). Therefore, the effects on gene expression are also greatly dependent on the probiotic strain, dose, bioactive compounds administered in combination, and the date and site of injection. Our results, however, highlight that the administration of the same probiotic can exert differential effects when combined with a phytobiotic. As different phytobiotics contain different biological components, this infers different effects on the host and it provides a wide range of opportunities for future research to elucidate more combinations for optimized effects on different genotypes of chickens.

## Conclusion

In conclusion, in ovo stimulation of broiler chickens with *Leuconostoc mesenteroides* B/00288 probiotic strain alone and in combination with 0.5% garlic aqueous extract (as a prophybiotic) display tissue-specific differential modulation of immune- and metabolic-related genes in immune-related organs. Our study demonstrated the possibility of combining a probiotic with a phytobiotic for in ovo application and encourages more research on prophybiotic combinations to optimize gene expression in broiler chickens.

## Data Availability

The datasets generated during and/or analyzed during the current study are available from the corresponding author on reasonable request.
